# Assortment of Frontiers in Protein Science

**DOI:** 10.3390/ijms23073685

**Published:** 2022-03-28

**Authors:** István Simon, Csaba Magyar

**Affiliations:** Institute of Enzymology, Research Centre for Natural Sciences, Eötvös Loránd Research Network, 1117 Budapest, Hungary

Recent decades have brought significant changes to the protein structure research field. Thanks to the genome projects and advances in structure determination methods, the number of yearly released entries in the PDB database [1] has increased significantly. Protein structure research is experiencing a new renaissance, and in 2020 the number of deposited structures in the PDB database reached a new record of 14,022. Even in 2021, the number of new deposits was higher than ever before, with the exception of 2020. Most of these structures belong to globular proteins, but there are several transmembrane and even disordered proteins among them [2]. Moreover, there are also transmembrane proteins with disordered regions that have led to the emergence of new transmembrane specific disorder prediction methods [3]. Additionally, we cannot forget to mention the huge leap forward in the field of structure prediction methods achieved by the AlphaFold2 [4] method, which is able to predict protein structures with an error comparable with that of experimental methods. Our Special Issue features a research article connected to this research area, which evaluates a deep learning-based residue contact method [5]. With the development of Alphafold-Multimer [6], the accurate prediction of protein complexes is becoming a reality. One important application of this method would be the prediction of protein–protein interactions. On the way to this goal, this Special Issue presents a work dealing protein–protein docking [7]. Of course, the COVID-19 pandemic has left inevitable traces on our lives over the last two years and also on research. Not only did the development of vaccines arrive to the frontier, but structure research of the severe acute respiratory syndrome coronavirus 2 (SARS-CoV-2) proteins became an intensively researched field. One such piece of research has made its way into this Special Issue, as well [8]. There is an additional research article in our issue with medical relevance that is about model development to predict the phenotypic outcome of rare germline pathogenic TP53 missense variants [9]. An assortment of many new frontiers is presented in this collection. A single issue cannot give a comprehensive overview of a large field such as proteins science, but we aim to give a broad overview of current research. In this issue, there are 19 research articles, one review, and one commentary. The manuscripts could have been categorized according to the subject of the research into these major categories like structure and the folding of globular proteins, membrane proteins, and disordered proteins or we could have classified them into theoretical and experimental groups. However, since several publications would fit into more than one category, we discarded this classification system. As manuscripts were published within a very short time upon acceptance, we decided to introduce the published papers is chronological order, starting with the latest one and concluding with the very first accepted manuscript of this Special Issue. Interestingly, both the first and last manuscripts deal with protein folding. We may conclude that protein folding is still the alpha and the omega of protein science. It is still the ultimate question, and even though research articles dealing with protein folding were published as long as 75 years ago this fundamental problem has yet to be solved. Several other manuscripts in this Special Issue deal with the folding problem, for example some deal with glycoproteins and disordered proteins. In the following paragraphs, the manuscripts published in our Special Issue *Frontiers in Protein Structure Research* are presented, which cover several fields of proteins science.

The fundamental problem of protein folding is reconsidered in the review of Sorokina et al. [10]. The generally accepted view of protein folding is the thermodynamic hypothesis, under which the native folded conformation of a protein corresponds to the global minimum of Gibbs free energy. The authors suggest that the evidence behind the thermodynamic hypothesis is not convincing. They argue that despite the continuous increase in computing power, only a few protein folds can be predicted by ab initio physics-based approaches. Furthermore, recent spectacular successes in protein structure prediction were achieved by deep learning-based evolution modeling methods. An alternative view of protein folding is proposed, implying that the native state of proteins lies in a local minimum of the fluctuating free energy surface. They also presume that the folding Gibbs free energy for numerous proteins is positive, and they are stabilized by the translation system and chaperones. Thus, folding should be modeled as a non-equilibrium energy-dependent in vivo process.

The conformational properties of covalently attached and bilayer contained carbohydrates influencing the structure of proteins were investigated by Guvench et al. [11] on a theoretical level. The ring puckering thermodynamics of the most common vertebrate monosaccharides were investigated by extended system-adaptive biasing force all-atom explicit-solvent molecular dynamics simulations. They found that the CHARMM [12] force field with proper parametrization is able to model the ring puckering of the investigated carbohydrates and could possibly be used widely for carbohydrate-containing vertebrate biomolecules. The accurate simulation of carbohydrates in glycoproteins, proteoglycans, and glycolipid-containing bilayer-embedded transmembrane proteins could help to narrow the gap in the number of suitable systems for theoretical and experimental methods and promote the in silico investigation of glycoproteins.

The possible structural background of the unusual behavior of mutual synergetic folding (MSF) proteins was analyzed by Magyar et al. [2]. These oligomeric proteins are disordered in their monomeric form but become almost completely ordered in their oligomeric form upon interacting with another disordered MSF protein chain. Solvent accessibility of the peptide bonds in the theoretical monomeric form seems to be a significant factor. Next to the local shielding effect of peptide bonds exerted by the side chains of the bond forming residues, nonlocal shielding also occurs upon oligomerization. To investigate these local and non-local shielding effects, Shannon information entropy calculations were performed on all available MSF and selected globular homodimeric proteins. According to the results, differences can be found in both local and non-local shielding. These findings open the possibility for a prediction method to distinguish MSF proteins from globular ones. The resulting larger dataset could be used to reveal the structural background of the MSF phenomenon.

The performance of the ProSPr distance prediction method, which is essentially an open-source alternative of the AlphaFold-1 contact prediction method, was evaluated by Stern et al. [5]. ProSPr is an accurate deep learning method to predict residue contacts based on amino acid sequence input. The authors tested the method on the CASP14 [13] test set and found that the ensemble predictions of short and mid contacts were reliable but that long contact prediction accuracy was only around 44%. They determined the useful multiple-sequence-alignment depth and found that amino acid sequence length did not correlate with contact prediction accuracy with the test set. The authors present a useful and accurate method with inference times two orders of magnitude faster than AlphaFold2. This tool could be helpful in many situations where the partial structural information of residue contacts is sufficient.

During the evolution of protein science, the discovery of transmembrane and later disordered proteins widened our view of the world of proteins. Dobson and Tusnady went one step further [3] and presented a novel method called MemDis, which is able to predict intrinsically disordered regions within transmembrane proteins. Although there are several protein disorder prediction methods, their accuracy is limited for membrane proteins, probably due to their special physicochemical properties. MemDis combines convolutional neural network and long short-term memory networks while adding transmembrane specific features to the prediction. The authors achieved an unprecedented level of disorder prediction accuracy on their transmembrane-specific test set. The method is publicly available at http://memdis.ttk.hu (accessed on 15 March 2022), providing an extremely useful tool for researchers to identify disordered regions within transmembrane proteins.

Phosphorylation-induced conformational change is a common way to regulate a protein’s function and disordered proteins are no exception. Rieloff and Skepö [14] investigated the impact of phosphorylation on the conformation of disordered proteins using molecular dynamics simulations. Since this a relatively new and under-researched field, first they validated the method by comparing the results obtained with two different force fields. While these force fields were known to overestimate the compactness of the phosphorylated state of disordered proteins mainly because of overstabilized salt bridges, they concluded that this discrepancy can be resolved with the proper incorporation of the effect of salt into the simulations, corresponding to the ionic strength present in the experiments. They found that the effect of salt concentration on simulation results is small enough to be neglected; thus, simulations can be used to help understand the mechanisms behind the phosphorylation regulation of disordered proteins. After publishing the results of this validation, Rieloff and Skepö published a second paper in this Special Issue. In their subsequent paper [15], they applied the previously validated Amber ff99SB-ILDN force field with the TIP4P-D water model and performed all-atom molecular dynamics simulations to analyze the effect of phosphorylation on five disordered peptides originating from tau, statherin, and beta-casein proteins. Their results were in qualitative agreement with the experimental data. They found that some peptides contracted upon phosphorylation while others became more expanded and that the amount of charges does not account for the phosphorylation-induced changes. The sequential distribution of residues with positive charges is crucial to describe this behavior through the formation of salt bridges with phosphorylated residues. They are conducting an ongoing systematic investigation of several factors influencing the outcome of phosphorylation.

The transmembrane region of HokC was investigated by Ortiz et al. [16] using a systematic saturation mutagenesis study. HokC is a toxin produced by *Escherichia coli* to control its own population. They found that 92% of the single-site point mutations were tolerated and that all the non-tolerated mutations had compensatory mutations that reversed their effect. By utilizing the HokC family multiple sequence alignment, they found only a single invariant cysteine residue. Every site-directed mutagenesis of this residue performed was also tolerated. The authors concluded that maintaining function without conserving amino acids is possible by compensatory mutations. Because of the helical transmembrane structure, sequentially close residues are expected to be close spatially. Thus, they may be suitable to accommodate compensatory mutations. Their findings were in agreement with this hypothesis, and the authors found that transmembrane proteins favor the occurrence of multiple mutations between spatially neighboring residues more than globular proteins. A notable exception is the mutation of the only invariant cysteine residue to serine, which causes a change in the dimerization of HokC. A complementary mutation occurred at sequentially distant positions, suggesting a change in interactions between different monomers.

The imaginary “smoking gun” by Bocedi et al. [17] is a provocative commentary, which presents experimental data with an unusual interpretation of the role of the glutathione tripeptide. They line up data on the hyper-reactivity of structural cysteines, the dependence of the second-order kinetic constants on pK_a_ values, and the reactivity of protein cysteines towards natural disulfides. Their interpretation may change our assumptions regarding the role of glutathione in the early steps of oxidative folding that occur at the ribosomal exit tunnel at the interface of the endoplasmic reticulum.

The problem of the structural dynamics of proteins was investigated by Nehls et al. [18] using conformation-sensitive oxidative protein labelling, which may serve as a complementary technique to mass spectrometry for capturing conformational changes. They used a test set of proteins between 10 and 150 kDa and showed that conformational changes induced by ligand binding are reflected in the modification of the mass spectrometry pattern obtained by site-selective Fenton chemistry labelling. For smaller proteins, the extensive oxidation pattern correlates well with the protein structural dynamics while there are clear differences between the oxidation patterns of the ligand-bound and free forms. Despite its practical limitations, this method could become a valuable tool for conformational analysis alongside mass spectrometry.

The toxicity of tetrabromobisphenol-S (TBBPS) was investigated by Jarosiewicz et al. [19], in order to see whether it would be a proper replacement for the widely used flame retardant tetrabromobisphenol-A (TBBPA), which is potentially toxic. They used red blood cell membranes as a model system. They found that both TBBPA and TBBPS caused increases in the fluidity of the membranes, decreases in the ATP level, thiol group elevation, and conformational changes to the membrane proteins. Both substances also caused changes in the size and shape of red blood cells and with TBBPS an increase in lipid peroxidation also occurred. They determined that changes are observed at significantly lower concentrations in the case of TBBPA than with TBBPS. The published data indicate lower toxicity for TBBPS, which occurs only at very high concentrations in contrast to TBBPA.

The thermodynamical properties of the SARS-CoV-2 virus spike protein variants were analyzed by Kumar et al. [8] using molecular mechanics and dynamics calculations in complex with the human ACE2 receptor. They performed molecular dynamics simulations to estimate the stability of the complex and calculated ΔG_bind_ binding free energy values using molecular mechanics calculations to characterize the strength of the binding. They found that the mutations caused stronger binding in the alpha and kappa variants. In the case of the kappa and delta variants, the mutations mainly increased the stability and intra-chain interactions in the spike protein, possibly interfering with the neutralizing effect of the antibodies, which might be responsible for the higher transmissibility of these variants.

Holubowicz et al. [20] identified single-nucleotide variants of the trimeric structure of globular C1q-like otolin-1, a collagen-like scaffold protein responsible for the biomineralization of inner ear stones in vertebrates. The globular-like gC1q-like domain binds calcium and is responsible for trimerization. The stability of the variants was analyzed by thermal shift assay and the positions of the mutated residues were mapped on a small angle X-ray scattering-derived model structure of the hOtolC1q trimer. According to the experiments, most of the mutations caused decreased stability or aggregation, but in most cases the structure can be stabilized in the presence of Ca^2+^. There is a Ca^2+^-insensitive a mutation that disables trimerization. The mean allele frequency of these deleterious mutations is in the range of 10^−4^. According to their results, these natural variants can cause pathological changes and affect one’s sense of balance.

The quaternary structure of the iota carbonic anhydrase (CA) from the marine diatom *Thalassiosira pseudonana* was modeled by Jensen et al. [21]. The protein is built up from domains resembling a calcium–calmodulin protein kinase II association domain. The crystal structure of the single domain was recently uncovered, and comparing it with available CA structures reveals novel folding element; however, the quaternary structure of the four domain-containing homotetrameric protein is still unknown. The authors utilized biophysical techniques and modelling to build the homotetrameric structure, which is formed from a core structure from the first two domains of each monomer, while the arms are formed by the other domains. The authors discussed the role of a flexible linker between domain 3 and 4 and a possible relation of its atypical shape with its activity and metal coordination. They also proposed a possible structure for carbonic anhydrases with fewer domain repeats using experimental data.

Bifidobacterial α-L-Fucosidases (ALF) were investigated by Curiel et al. [22], which are important for the bifidobacterial colonization of the gut. Several ALFs have been identified by bioinformatical methods, which can be classified into three major families. Bifidobacterial ALFs show significant sequential differences, probably resulting from distinct phylogenetic evolution. The authors performed phylogenetic and comparative analyses of bifidobacterial ALFs utilizing existing physicochemical information. They revealed several ALF paralogue groups within two major ALF families. The authors suggest that because ALFs are phylogenetically related to other glycosyl hydrolase families they may exhibit additional glycosidase activities that utilize transfucosylate substrates other than lactose. This could have a substantial impact on the development of novel prebiotics.

The nuclear factor erythroid 2-related factor 2 (Nrf2) was studied by Karunatilleke et al. [23]. Nfr2 can interact with several proteins and mediates the transcription of cytoprotective genes in cellular responses to oxidative stress. Nrf2 is a promising target for anticancer drug design, but the limited information about its molecular details and interactions hinder rational drug design. The authors applied combined bioinformatics with experimental methods like CD and NMR spectroscopy approaches to characterize the structure of Nrf2, and hydrogen deuterium exchange mass spectrometry was used to analyze its interaction with the Kelch domain of an interaction partner. They found that Nrf2 is partially disordered with transiently ordered segments. Binding with the Kelch domain stabilizes the structure of other binding motifs while leaving other regions highly dynamic. According to their results, the conformational dynamics of full length Nrf2 have substantial consequences for its target recognition, enabling Nrf2 to bind to distinct targets with high specificity and low affinity.

Wesch et al. [24] examined the UFM1-activating enzyme 5 (UBA5) within the ufmylation cascade of the ubiquitin fold modifier 1 (UFM1) protein. This cascade reaction affects several cellular processes and plays a role in the pathogenicity of many human diseases, but the molecular mechanisms of the ufmylation cascade are still unclear. The authors focused on the biophysical and biochemical characterization of the interaction between UBA5 and the UFM1-conjugating enzyme 1 (UFC1). Their working hypothesis was that the unstructured C-terminal of UBA5 regulates the cellular localization of the elements in the ufmylation cascade. According to their results, the C-terminal 20 residues in UBA5 are crucial for UFC1 binding. They uncovered the NMR structure of UFC1 complexed with this C-terminal peptide and identified key residues in the UBA5–UFC1 interaction. The structural evidence augmented with isothermal titration calorimetry results revealed the mechanism of the interaction and confirmed the crucial role of the C-terminal unstructured region.

A novel protocol for protein–protein docking was developed by Kurcinski et al. [7], incorporating protein–protein orientation and backbone flexibility with a single simulation step instead of the traditional two-step procedure. Exhaustive sampling is required for this approach, which can be achieved using the CABS coarse-grained protein model and replica exchange Monte Carlo dynamics. In this proof of concept study, the new protocol was tested on 62 protein–protein complexes. They found that the modeling of large conformational changes was possible with acceptable computational costs within the range of 10 CPU hours. For low- and medium-flexibility cases, the acceptable accuracy can be achieved with an iRMSD of around 4 Å, but the selection of the most accurate model needs to be improved with a success rate of only around 50%. The current common approaches to taking flexibility into account have serious limitations. The proposed protocol is conceptually different and relies heavily on the exhaustive sampling capability of the simplified simulations, which is orders of magnitude faster than classical force field-based molecular dynamics simulations. While these kinds of simulations also have their limitations when reproducing the real free energy surface, the more exhaustive sampling could compensate for their weaknesses. Although this protocol opens new perspectives in flexible protein–protein docking applications, it also has limitations. Despite the high performance of the replica exchange annealing enhanced Monte Carlo dynamics, this method still scales poorly with the size of the “ligand” proteins setting a practical limit of about 150 residues. The simplified description of atomic interaction forces results in less sensitive docking energetics, which makes the above-described selection of the most accurate model difficult. The authors suggest that parallelizing the simulation may speed up the process. In this way, an automated public protein–protein docking server could be created, which could be a very useful tool for studying protein–protein interactions.

A logistic regression model was developed by Liu et al. [9] in order to predict the phenotypic outcome of rare germline pathogenic TP53 missense variants. They compiled non-overlapping datasets for the Li-Fraumeni syndrome and hereditary breast cancer outcomes. TP53 protein is a transcription factor that binds as a tetramer to DNA and activates a large number of genes that promote DNA repair mechanisms or apoptosis. Each monomer is built up from several domains, including a DNA binding domain and an oligomerization domain, among others. About two-thirds of reported germline TP53 variants are single-site missense changes. Predominantly located in the DNA binding domain, some of them result in the decreased thermal stability of this domain. By utilizing an X-ray structure of TP53, the conformational characteristics of the variants were included in the method. The models show a clear relationship between disease outcome for TP53 variants and their effects on aspects of protein conformation and function. The model could be helpful to avoid unnecessary examinations for a large proportion of TP53 variant carriers, which could relieve pressure on the medical system.

Pressure denaturation of the all-α GH2 domain of the GIPC1 protein adaptor was investigated using NMR spectroscopy by Dubois et al. [25]. To date, this method has been used mainly for small α/β and all-β single domain proteins. High-pressure perturbation was used with NMR spectroscopy to reveal the unfolding landscape at 10, 20, and 30 °C, and the results were compared with chemical denaturation experiments. While GIPC1-GH2 is most stable at 20 °C, it is more stable at 30 °C than at 10 °C. Their finding that the loss of tertiary and secondary structure was quasi-simultaneous was unexpected, meaning that helices are not stable outside the 3D scaffold. The unfolding was cooperative at high pressures and the highest temperatures but more progressive at the lowest temperatures. Although partial unfolding can occur at lower temperatures, at 30 °C the stability is decreased and thermal denaturation probably competes with high-pressure denaturation, sweeping away the partial unfolding that occurs at lower temperatures. The authors demonstrated the usefulness of pressure-induced unfolding experiments in exploring the unfolding landscape of proteins by monitoring the partial unfolding process, which could not have been followed by chemical denaturation.

Finally, we arrive at the first published paper in our Special Issue by Liu et al. [26], which emphasizes the importance of considering conformational entropy accurately for the simulation of disordered proteins. There are several pairwise additive force fields that were specifically modified to handle disordered proteins more accurately, yet they still often fail to reproduce experimental results. The authors propose the incorporation of configurational entropy for the development of universal force fields, which should be able to handle globular and disordered proteins and disorder to order transitions equally well. They compared pairwise additive force fields with the AMOEBA [27] many-body force field using experimental data on a set of disordered and medium-sized globular proteins. According to their results, fixed-charge force fields gave smaller yields, while the polarizable model yielded larger RMSD for ordered proteins. Force fields with the largest RMSD fluctuations are consistent with the results from the radius of gyration experiments. They argued that by exhibiting larger variations, they are better suited to describe the structural plasticity of disordered proteins. According their results, the polarizable AMOEBA many-body force field is beneficial for the simulation of disordered proteins and it can outperform specifically modified force fields without requiring problem-specific parametrization. By retaining its universality, it is well suited as a general force field for different types of disordered proteins and their complexes. They suggest, however, that in their evaluation the precision of the examined pairwise additive force fields was not adequate and that further efforts to reproduce the structural dynamics could be used as guidance for the development and validation of force fields. They concluded that force fields with the largest variations in the radius of gyration and universal Lindeman values for folded states describe disordered proteins and disorder to order transitions better and that a universal force field applicable to globular and disordered proteins should be able to describe the balance between energetics and configurational entropy.

In this Special Issue, we aim to represent the vibrant state of protein structure studies at the end of 2021 and the flowering of this field since the middle of the nineteenth century with this assortment of publications. The editors hope that the readers will welcome it!

## References

[1] Berman H.M., Westbrook J., Feng Z., Gilliland G., Bhat T.N., Weissig H., Shindyalov I.N., Bourne P.E. (2000). The Protein Data Bank. Nucleic Acids Res..

[2] Magyar C., Mentes A., Cserző M., Simon I. (2021). Origin of Increased Solvent Accessibility of Peptide Bonds in Mutual Synergetic Folding Proteins. Int. J. Mol. Sci..

[3] Dobson L., Tusnády G.E. (2021). MemDis: Predicting Disordered Regions in Transmembrane Proteins. Int. J. Mol. Sci..

[4] Jumper J., Evans R., Pritzel A., Green T., Figurnov M., Ronneberger O., Tunyasuvunakool K., Bates R., Žídek A., Potapenko A. (2021). Highly accurate protein structure prediction with AlphaFold. Nature.

[5] Stern J., Hedelius B., Fisher O., Billings W.M., Della Corte D. (2021). Evaluation of Deep Neural Network ProSPr for Accurate Protein Distance Predictions on CASP14 Targets. Int. J. Mol. Sci..

[6] Evans R., O’Neill M., Pritzel A., Antropova N., Senior A.W., Green T., Žídek A., Bates R., Blackwell S., Yim J. (2021). Protein Complex Prediction with AlphaFold-Multimer. https://www.biorxiv.org/content/10.1101/2021.10.04.463034v2.

[7] Kurcinski M., Kmiecik S., Zalewski M., Kolinski A. (2021). Protein–Protein Docking with Large-Scale Backbone Flexibility Using Coarse-Grained Monte-Carlo Simulations. Int. J. Mol. Sci..

[8] Kumar V., Singh J., Hasnain S.E., Sundar D. (2021). Possible Link between Higher Transmissibility of Alpha, Kappa and Delta Variants of SARS-CoV-2 and Increased Structural Stability of Its Spike Protein and hACE2 Affinity. Int. J. Mol. Sci..

[9] Liu Y., Axell O., van Leeuwen T., Konrat R., Kharaziha P., Larsson C., Wright A.P.H., Bajalica-Lagercrantz S. (2021). Association between Predicted Effects of *TP53* Missense Variants on Protein Conformation and Their Phenotypic Presentation as Li-Fraumeni Syndrome or Hereditary Breast Cancer. Int. J. Mol. Sci..

[10] Sorokina I., Mushegian A.R., Koonin E.V. (2022). Is Protein Folding a Thermodynamically Unfavorable, Active, Energy-Dependent Process?. Int. J. Mol. Sci..

[11] Guvench O., Martin D., Greene M. (2022). Pyranose Ring Puckering Thermodynamics for Glycan Monosaccharides Associated with Vertebrate Proteins. Int. J. Mol. Sci..

[12] Sairam S., Mallajosyula S., Guvench O., Hatcher E., MacKerell A.D. (2012). CHARMM Additive All-Atom Force Field for Phosphate and Sulfate Linked to Carbohydrates. J. Chem. Theory Comp..

[13] Kryshtafovych A., Schwede T., Topf M., Fidelis K., Moult J. (2021). Critical assessment of methods of protein structure prediction (CASP)-Round XIV. Proteins.

[14] Rieloff E., Skepö M. (2021). Molecular Dynamics Simulations of Phosphorylated Intrinsically Disordered Proteins: A Force Field Comparison. Int. J. Mol. Sci..

[15] Rieloff E., Skepö M. (2021). The Effect of Multisite Phosphorylation on the Conformational Properties of Intrinsically Disordered Proteins. Int. J. Mol. Sci..

[16] Lara Ortiz M.T., Martinell García V., Del Rio G. (2021). Saturation Mutagenesis of the Transmembrane Region of HokC in *Escherichia coli* Reveals Its High Tolerance to Mutations. Int. J. Mol. Sci..

[17] Bocedi A., Cattani G., Gambardella G., Schulte L., Schwalbe H., Ricci G. (2021). Oxidative Folding of Proteins: The “Smoking Gun” of Glutathione. Int. J. Mol. Sci..

[18] Nehls T., Heymann T., Meyners C., Hausch F., Lermyte F. (2021). Fenton-Chemistry-Based Oxidative Modification of Proteins Reflects Their Conformation. Int. J. Mol. Sci..

[19] Jarosiewicz M., Duchnowicz P., Jarosiewicz P., Huras B., Bukowska B. (2021). An In Vitro Comparative Study of the Effects of Tetrabromobisphenol A and Tetrabromobisphenol S on Human Erythrocyte Membranes—Changes in ATP Level, Perturbations in Membrane Fluidity, Alterations in Conformational State and Damage to Proteins. Int. J. Mol. Sci..

[20] Hołubowicz R., Ożyhar A., Dobryszycki P. (2021). Natural Mutations Affect Structure and Function of gC1q Domain of Otolin-1. Int. J. Mol. Sci..

[21] Jensen E.L., Receveur-Brechot V., Hachemane M., Wils L., Barbier P., Parsiegla G., Gontero B., Launay H. (2021). Structural Contour Map of the Iota Carbonic Anhydrase from the Diatom *Thalassiosira pseudonana* Using a Multiprong Approach. Int. J. Mol. Sci..

[22] Curiel J.A., Peirotén Á., Landete J.M., Ruiz de la Bastida A., Langa S., Arqués J.L. (2021). Architecture Insight of Bifidobacterial α-L-Fucosidases. Int. J. Mol. Sci..

[23] Karunatilleke N.C., Fast C.S., Ngo V., Brickenden A., Duennwald M.L., Konermann L., Choy W.-Y. (2021). Nrf2, the Major Regulator of the Cellular Oxidative Stress Response, is Partially Disordered. Int. J. Mol. Sci..

[24] Wesch N., Löhr F., Rogova N., Dötsch V., Rogov V.V. (2021). A Concerted Action of UBA5 C-Terminal Unstructured Regions Is Important for Transfer of Activated UFM1 to UFC1. Int. J. Mol. Sci..

[25] Dubois C., Planelles-Herrero V.J., Tillatte-Tripodi C., Delbecq S., Mammri L., Sirkia E.M., Ropars V., Roumestand C., Barthe P. (2021). Pressure and Chemical Unfolding of an α-Helical Bundle Protein: The GH2 Domain of the Protein Adaptor GIPC1. Int. J. Mol. Sci..

[26] Liu M., Das A.K., Lincoff J., Sasmal S., Cheng S.Y., Vernon R.M., Forman-Kay J.D., Head-Gordon T. (2021). Configurational Entropy of Folded Proteins and Its Importance for Intrinsically Disordered Proteins. Int. J. Mol. Sci..

[27] Shi Y., Xia Z., Zhang J., Best R., Wu C., Ponder J.W., Ren P. (2013). The Polarizable Atomic Multipole-based AMOEBA Force Field for Proteins. J. Chem. Theory Comput..

